# What do Australian & New Zealand caregivers know about children’s ibuprofen? The results of an online survey?

**DOI:** 10.1186/s12887-018-1297-2

**Published:** 2018-10-15

**Authors:** Judy Mullan, Pippa Burns, Kathryn Weston, Shelley Crowther, Robyn Dixon, Emma Moselen

**Affiliations:** 1 Centre for Health Research Illawarra Shoalhaven Population (CHRISP), University of Wollongong, Wollongong, NSW 2522 Australia; 2 School of Medicine, University of Wollongong, Wollongong, Australia; 3 The Pharmaceutical Society of Australia, Royal Parade, Parkville Vic, 3053 Australia; 4 School of Nursing, University of Auckland, Auckland, 1142 New Zealand

**Keywords:** Ibuprofen, Children, Caregivers, Knowledge, Adverse drug events

## Abstract

**Background:**

Children’s formulations containing ibuprofen are frequently used to treat pain and fever. These medications, available over-the-counter, have the potential to cause adverse events if usage/safety information is not adhered to. This study aimed to investigate caregivers’ knowledge about the safe use of a commonly purchased children’s formulation containing ibuprofen.

**Methods:**

A convenience sample of caregivers in Australia and New Zealand, who had purchased *Nurofen® for Children,* completed an online survey assessing their knowledge of product information and dosage instructions available on/in the product packaging.

**Results:**

In total, 219 caregivers (mainly female 95%, mean age ± SD; 35 ± 6.82 years) completed the online survey. Responses suggest limitations in their knowledge about the active ingredients, contraindications and side effect profile associated with the product. Most respondents had a good understanding about the correct dosage to give children based on their weight and/or age, but many lacked a good understanding about the correct interval between doses and the maximum number of daily doses.

**Conclusions:**

These findings suggest that caregivers administering ibuprofen to children have gaps in their product knowledge. Strategies to help improve caregivers’ knowledge about the safe administration of these products should be prioritised in an attempt to reduce the likelihood of children experiencing ibuprofen related adverse events. Improving caregiver knowledge to address these gaps is an important issue for all health care providers.

**Electronic supplementary material:**

The online version of this article (10.1186/s12887-018-1297-2) contains supplementary material, which is available to authorized users.

## Background

Ibuprofen, a non-steroidal anti-inflammatory drug (NSAID) commonly used to treat pain and fever in children, is readily available for purchase over-the-counter (OTC) [1]. While ibuprofen is considered to be a safe drug to use in children [2, 3], it can be associated with adverse events, especially if the dosage instructions are not adhered to [4]. The most commonly reported ibuprofen related adverse events include gastro-intestinal, renal and respiratory complications, with dehydration playing an important role in triggering renal complications [1, 2, 5].

Important product information, precautions for use and dosage instructions can generally be found on or within the product packaging of children’s formulations containing ibuprofen**.** However, irrespective of this available information, ibuprofen continues to be one of the main contributors to drug related adverse events [1, 6, 7], and hospitalisation [8] in children.

## Methods

This study sought to investigate caregivers’ knowledge about the safe use of *Nurofen® for Children*. This brand was specifically chosen for this study because it is a well-recognised children’s ibuprofen formulation available for purchase in many different countries.

Ethics approval was granted by the University of Wollongong’s Human Research Ethics Committee (HE14/455) and the University of Auckland’s Human Participants Ethics Committee (014682).

A cross-sectional online survey (Additional file 1) was administered via Survey Monkey between April and November 2015. The 18 survey questions included: five demographic questions (including a single item literacy screening question [9]); four questions about which product had been purchased, from where and for what purpose, as well as the age(s) of the child(ren) for whom the products had been purchased; and seven product knowledge questions related to the safe administration of *Nurofen® for Children* (answers were available on the product packaging). To assist with answering the final two dosage related questions [If your child was 8 months old, what dose of *Nurofen® for Children* 1–5 years should you give?, and If your child weighed 20 kg, what dose of *Nurofen® for Children* 1–5 years should you give?], participants were provided with the information in Table 1.

**Table 1:** Product information taken from product packaging of *Nurofen® for Children* 1–5 years

Age	Average weight	Dose
3–6 months	6–8 kg	3–4 mL
6–12 months	8–10 kg	4–5 mL
1–3 years	10–14 kg	5–7 mL
3–5 years	14–18 kg	7–9 mL
5–7 years	18–22 kg	9–11 mL
7–9 years	22–28 kg	11–14 mL
9–12 years	28–40 kg	14–20 mL

A convenience sample of adult caregivers in Australia and New Zealand were recruited through promotion of the survey via Facebook, organisation newsletters (such as Plunket and Mainly Music), as well as via parents’ coffee and play groups. The link to the survey was shared with potential participants, who were provided with participant information which explained why the study was being conducted, who the investigators were and how long the survey would take to complete. Potential participants were informed at the beginning of the survey that they needed to be ≥18 years of age and have previously purchased one or more of the following products: *Nurofen® for Children* Baby 3+ months (200 mg of ibuprofen per 5 mL); *Nurofen® for Children* 1–5 years (100 mg ibuprofen per 5 mL); and *Nurofen® for Children* 5–12 years (200 mg ibuprofen per 5 mL).

Data were downloaded from Survey Monkey and stored on a password protected desk-top computer. Data were analysed using the Statistical Package for the Social Sciences (SPSS v.21) and reported according to the CHERRIES guidelines [10].

## Results

The majority of the 219 respondents were English speaking (*n* = 211, 96.3%), female (*n* = 207, 94.5%), and aged between 22 to 67 years (mean age ± SD; 35 ± 6.82 years). Most respondents had a university qualification (*n* = 169, 77.2%), and adequate functional health literacy scores (*n* = 216, 98.6%).

*Nurofen® for Children* was almost exclusively purchased from a pharmacy (*n* = 214, 97.7%) for the treatment of fever (*n* = 145, 66.2%) and pain (*n* = 120, 54.8%). The average age of the children for whom the products were purchased was 4.3 ± 3.55 years. The following is a breakdown of the products purchased by respondents, noting that some may have purchased more than one product: *Nurofen® for Children* Baby 3+ months (*n* = 88, 40.2%); *Nurofen® for Children* 1–5 years (*n* = 104; 47.5%): and *Nurofen® for Children* 5–12 years (*n* = 75, 34.2%).

### Knowledge about Nurofen® for children

Most respondents knew that *Nurofen® for Children* contained ibuprofen (*n* = 198, 90.4%). However, many respondents incorrectly believed that the product contained; paracetamol (*n* = 64, 29.2%), alcohol (*n* = 56, 25.6%), codeine (*n* = 49, 22.3%) and/or aspirin (*n* = 48, 21.9%).

As seen in Table 2, the majority of the respondents knew to seek medical advice from a doctor or a pharmacist, prior to giving *Nurofen® for Children* to children who: were taking other medications (93.2%); suffered from asthma (81.3%); or were under 12 months of age (77.6%). The vast majority correctly identified that *Nurofen® for Children* was contraindicated in children with an ibuprofen allergy (91.3%). However, their knowledge that an aspirin allergy was also a contraindication and that allergies/intolerance to milk products, eggs and gluten were not contraindications was quite poor. In addition, respondents’ knowledge of potential side effects associated with *Nurofen® for Children* overdose was variable. The majority knew that overdose could cause stomach problems (77.6%); however, less than half recognised that kidney problems could eventuate. In addition, only 5% recognised ringing in the ears as a possible side effect, and just over one-in-five respondents knew that that liver poisoning (22.4%) was not a potential side effect.

**Table 2:** Correct responses for possible contraindications or side effects

Survey questions	Number of correct responses	Percentage of correct responses (%)
Should you check with your doctor or pharmacist before giving *Nurofen® for Children*…(correct answer in brackets)		
If they are on other medications (yes)	204	93.2
If they have asthma (yes)	178	81.3
If they are aged under 12 months (yes)	170	77.6
You can give *Nurofen® for Children* to children who are…		
Allergic to ibuprofen (no)	202	91.3
Allergic to aspirin (no)	62	28.3
Allergic to milk products (yes)	88	40.2
Allergic to eggs (yes)	80	36.5
Gluten intolerant (yes)	76	34.7
Which of the following side effects might be experienced when given too much *Nurofen® for Children*		
Stomach problems (yes)	170	77.6
Kidney problems (yes)	104	47.5
Liver poisoning (no)	49	22.4
Ringing in the ears (yes)	11	5.0

### Comprehension of dosage instructions

Over three-quarters of respondents knew that *Nurofen® for Children* could be safely given for up to three consecutive days (*n* = 165, 75.3%). Fewer than half of them (*n* = 107, 48.9%) correctly identified that a six-hour interval was the minimum recommended time between doses and a similar proportion knew that three doses is the maximum a child should receive in a 24-h period (*n* = 94, 42.9%). Based on the responses to the two questions regarding the correct dosage of *Nurofen® for Children* 1–5 years for an 8 month old child, and a child who weighed 20 kg, the majority of respondents correctly interpreted the dosage instructions in both scenarios (*n* = 194, 88.6%; and *n* = 193, 88.1% respectively).

## Discussion

While the majority of participants in the current study were able to calculate dosage correctly, based on the age and/or the weight of the child, it is concerning that many of them had limitations in their knowledge about the safe use of *Nurofen® for Children*. Similar gaps in knowledge were also reported in a study focusing on an adult ibuprofen formulation [11]. The lack of understanding of the contraindications and side effects in this study are also concerning, as they indicate that children may be placed at potential risk during medically unsupervised care at home. Further, given that the respondents were, in the main, highly educated English speaking females with adequate functional health literacy, it is likely that our result underestimates the true level of understanding in the wider population. Moreover, since, *Nurofen® for Children* is frequently purchased to treat fever it is quite possible that many caregivers are unaware that dehydration, potentially exacerbated by fever, may elicit ibuprofen related renal complications [1, 2]. Further, a study of 78 caregivers of children aged 0–10 years, found that 42.3% admitted to using ibuprofen more frequently than recommended [12]. Thus, the potential for harm to children may be further compounded, explaining in part, recent increases in ibuprofen related adverse events [1].

Furthermore, whilst the majority of respondents were correctly able to identify ibuprofen as an active ingredient in *Nurofen® for Children*, many of them incorrectly believed that paracetamol, alcohol and/or codeine were also active ingredients. This lack of understanding, together with their beliefs that the product could not be administered to children with certain food allergies/intolerances, may result in children not receiving adequate treatment to relieve their symptoms.

### Limitations

Given the relatively small sample size comprising of  highly educated females, the focus on a specific brand of children’s ibuprofen and the self-reported nature of the survey, there are limitations to the generalisability of the results.

## Conclusions

These findings suggest that caregivers have gaps in their knowledge about the safe use of children’s formulation containing ibuprofen. Strategies to address these knowledge gaps need to be developed and we suggest should include: improving product labelling; improving communication between pharmacists and caregivers; as well as the provision of good quality information available in the media and online. In view of the ready access of these OTC products and our belief that the results of this study are probably an underestimation of the true level of the problem, there is some urgency required to ensure caregivers’ knowledge of these products is improved.

## Additional file

Additional file 1: Participant information sheet and Survey questions. (PDF 116 kb)
