# Supplementary material for: What do Australian & New Zealand caregivers know about children’s ibuprofen? The results of an online survey?
Source: BMC Pediatr. 2018 Oct 15;18:327. doi: 10.1186/s12887-018-1297-2 (PMC6190653; doi:10.1186/s12887-018-1297-2)
Supplement: Supplementary file 1 — Participant information sheet and Survey questions. (PDF 116 kb) [file 12887_2018_1297_MOESM1_ESM.pdf]

University of Wollongong, Graduate School of Medicine

PARTICIPANT INFORMATION SHEET

TITLE: How well do parents and carers understand the information on "Nurofen for Children" medication packaging?

PURPOSE OF THE RESEARCH

This is an invitation to participate in a study to investigate people's understanding of the instructions on the packaging of 'Nurofen for Children'. We want to find out if the information on the packaging is the right kind of information for people to safely use these medicines.

METHOD AND DEMANDS ON PARTICIPANTS

If you choose to be included in this study, you will be asked some information about yourself and how well you understand the information on the packaging of the 'Nurofen for Children' product you purchased and answer some questions about the dose and effects of the medication. All information will be kept confidential and your name will not be identified in any reports or publications arising from this research.

POSSIBLE RISKS, INCONVENIENCES AND DISCOMFORTS

The survey should take about 10 to 20 minutes to complete. Apart from the time taken to complete the survey, we can foresee no risks for you. Your involvement in the study is voluntary and refusal to participate in the study will not affect your relationship with your pharmacist or the university.

OUTCOMES OF THE RESEARCH

The investigators will analyse the data from all the participants and publish the results in scientific and/or medical journals and present the findings at scientific and/or medical conferences. By completing the survey you are giving consent that the information you provide can be used in this way. Results of this research project will be used to better understand if the information on the packaging is the right information for people to safely use these medicines.

ETHICS REVIEW AND COMPLAINTS

This study has been reviewed by the Human Research Ethics Committee of the University of Wollongong and the University of Auckland. If you have any concerns or complaints regarding the way this research has been conducted, you can contact the Research Services Office - Ethics Unit at rso-ethics@uow.edu.au.

Thank you for your interest in this study. If you would like further information about this study please contact the chief Investigator A/Prof Judy Mullan at jmullan@uow.edu.au

1. Which medicine did you buy?

☐ Nurofen® for Children Baby 3+ months
☐ Nurofen® for Children 1-5 years
☐ Nurofen® for Children 5-12 years

Other (please specify)

2. Where did you purchase this medicine?

☐ From a pharmacy
☐ Online

Other (please specify)

3. How old is the child and/or children who will be receiving this medication?

	Years	months
Age of Child 1		
Age of Child 2		
Age of Child 3		
Age of Child 4		
Age of Child 5		

Other (please specify)

4. Why did you buy this medicine? Please tick as many responses as are appropriate.

☐ Pain
☐ Teething
☐ Cold/flu
☐ Sleep
☐ Fever
☐ Earache

Other (please specify)

5. What ingredients are in this medicine? Please choose YES, NO or UNSURE for each option.

	Yes	No	Unsure
Paracetamol	☐	☐	☐
Aspirin	☐	☐	☐
Ibuprofen	☐	☐	☐
Alcohol	☐	☐	☐
Codeine	☐	☐	☐

6. When should you check with your doctor or pharmacist before giving this medicine to children? Please choose YES, NO or Unsure for each option.

	Yes	No	Unsure
If they are under 12 months of age	☐	☐	☐
If they have asthma	☐	☐	☐
If they are on other medicine	☐	☐	☐

7. Can you give this medicine to children who are…
Please choose YES, NO or Unsure for each option.

	Yes	No	Unsure
Allergic to ibuprofen?	☐	☐	☐
Allergic to aspirin?	☐	☐	☐
Allergic to milk products?	☐	☐	☐
Allergic to eggs?	☐	☐	☐
Gluten intolerant.	☐	☐	☐

8. Which of the following side effects might be experienced by children given too much of this medicine. Please tick as many answers as you think are side effects.

☐ Kidney problems
☐ Liver poisoning
☐ Ringing in the ears
☐ Stomach problems

9. What is the MAXIMUM amount of this medicine that a child can have in one day (24 hours)? Please tick ONE answer only.

☐ 1 dose
☐ 2 doses
☐ 3 doses
☐ 4 doses
☐ Up to six doses

Other (please specify)

10. How many days in a row can you safely give this medicine to a child? Please tick ONE answer only.

☐ Up to 3 days
☐ Up to a week
☐ Up to a month
☐ Up to 6 months
☐ Up to 12 months or longer

11. If you give a dose of this medicine to a child at 8 am, when is the earliest time that another dose can be given on the same day? Please tick ONE answer only.

☐ At 10.00 am
☐ At 12:00 noon
☐ At 2.00 pm
☐ At 4:00 pm

Please read the sample information provided from the packet of the Nurofen® for Children 1-5 and answer the following questions.

12. If your child was 8 months old, what dose of ‘Nurofen® for Children 1-5 years’ do you think you should give? Please tick ONE answer only.

☐ 3 - 4 mL
☐ 4 – 5mL
☐ 5 – 7mL
☐ 7 – 9 mL
☐ 9 -11 mL

13. If your child weighed 20 kilogram what dose do you think you should give? Please tick ONE answer only.

☐ 5-7 mL
☐ 7- 9mL
☐ 9 – 11 mL
☐ 11- 14 mL
☐ 14 – 20mL

About You

14. How old are you?

15. What is your sex?

☐ Male
☐ Female

16. What language do you usually speak at home?

17. What is the highest level of education you reached? Please tick ONE box.

- ☐ Primary school or Year 6 and below
- ☐ Junior high school or Years 7 to 10
- ☐ Senior high school or Years 11 and 12
- ☐ TAFE or trade certificate
- ☐ College or University

18. How often do you need to have someone help you when you read instructions, pamphlets or other written material from your doctor or pharmacy? Please tick ONE box

- ☐ All of the time
- ☐ Most of the time
- ☐ Some of the time
- ☐ A little of the time
- ☐ None of the time

Other (please specify)

THANK YOU

If you would like to enter a competition for a $200 Coles Myer voucher, please provide us with your contact details below (email address or telephone number).

If you have any questions or require information relating to the use of Nurofen® for your child you can speak to your pharmacist or doctor. More information about Nurofen® is available at http://www.nurofen.com.au/infants-children

If you wish to enter the draw for the Coles Myer voucher please enter details below (email or telephone number).
